# What Will the Veterinarians of Illinois Do?

**Published:** 1898-06

**Authors:** 


### EDITORIAL.

### WHAT WILL THE VETERINARIANS OF ILLINOIS DO?

The need of one strong movement of the veterinarians over the land for recognition with rank of our army veterinarians will bring the desired end. Of the thousands of men to-day with their respective regiments in active service, the veterinarian stands alone as one who cannot look for promotion, emoluments, or provisions for wounds, disabilities, or his family's needs in case of his death. Two regimental veterinarians have been killed in active service on the frontier, and their families were allowed to suffer by a wealthy government, while the widows and children of the officers and privates are bountifully provided for. Two of the present army veterinarians now en route for Cuba have a record of over thirty-five years' service, and ought to be at home enjoying their old age with their families on a liberal pension instead of being forced by necessity to encounter hardships and privations which even young men naturally dread.

In addition, there are over twenty thousand valuable animals in active service without any veterinary attendance.

Surely this state of affairs should not exist any longer. Spain had her commissioned veterinarians in our country during the past six months selecting horses and mules, and our own country stands alone in the civilized world with such a state of affairs existing.

The people of our country do not mean that this state of affairs should exist, and if every veterinarian will now do his duty it will end this unjust reflection on our profession. Pennsylvania will raise two hundred dollars at once for the purpose of making battle for our colleagues in the army. Illinois is strong in good earnest members of the profession, strong in college and association circles, and we ask of her six hundred and more devotees of the profession which this issue of the Journal reaches, WHAT WILL HER VETERINARIANS DO?

